# An unusual finding of schwannoma in the mammary gland of a dog

**DOI:** 10.17221/41/2024-VETMED

**Published:** 2024-10-30

**Authors:** Maria Makovicka, Peter Bolgac, Karol Kajo, Peter Makovicky, Pavol Svorc

**Affiliations:** ^1^Department of Histology and Embryology, Faculty of Medicine, University of Ostrava, Ostrava, Czech Republic; ^2^Cancer Research Institute, Biomedical Research Centre of the Slovak Academy of Sciences, Bratislava, Slovak Republic; ^3^Department of Pathology, Veterinary and Food Institute, Bratislava, Slovak Republic; ^4^Department of Pathology, St. Elisabeth Cancer Institute, Bratislava, Slovak Republic; ^5^Infectious Diseases and Preventive Medicine, Veterinary Research Institute, Brno, Czech Republic; ^6^Institute of Physiology and Pathophysiology, Faculty of Medicine, University of Ostrava, Ostrava, Czech Republic

**Keywords:** histopathology, hybrid tumours, mammary gland, perineural cells, peripheral nerve cells, Schwann cells, veterinary oncology

## Abstract

Peripheral nerve sheath tumours (PNSTs) are rare in the mammary glands of dogs. Here, we report a case of a schwannoma, composed of two parts, of the mammary gland of a dog. The first part consists of clusters of uncircumscribed, alternating, more concentrated and looser regions. In the more concentrated parts, typical fascicularly arranged schwannoma intermingle in plexiform arrangement, more subtle in appearance than in neuronal tumour areas. The second part undergoes regression consisting predominantly of residual shorter rosettes of cells with the presence of a peculiar variably sized swirling of target-like formations consisting of compact, thicker, pinkish lamellae also with occasional adjacent cells. Immunohistochemically, the tumour cells are positive for evidence of vimentin and neuro-specific enolase. They exhibit the variable expression of the S-100 protein, show mild CD56 positivity, and focally mildly accentuated proliferative activity as assessed by Ki-67. The tumour elements are negative for evidence of cytokeratin 7, cytokeratin 20, and oestrogen receptors. Hybrid tumours may change their morphology in combination with atypical localisation and may be underdiagnosed in veterinary biopsy practice. They differ from epithelial tumours prognostically, as well as in their development and behaviour, therefore it is essential to clearly differentiate them.

Tumours arising from peripheral nerves are divided into two separate categories according to the World Health Organization (WHO) classification of skin and soft tissue tumours (Maxie 2016; Hendrick 2017), namely benign tumours, which include traumatic neuroma, granular cell tumours, and benign peripheral nerve sheath tumours of the skin and subcutis (BPNSTs). The malignant form is represented by malignant peripheral nerve sheath tumours of the skin and subcutis (MPNSTs). If we define the group of peripheral nerve sheath tumours (PNSTs), they are tumours arising from Schwann cells, perineural cells, which can be organised into the typical BPNSTs characteristic of the schwannoma, perineurioma, neurofibroma, or MPNSTs, to which neurofibrosarcoma and malignant schwannoma belong. Within PNSTs, these have been described in several animals with the most common localisation of occurrence in the nervous system, but also outside the peripheral and central nervous system (Texeira et al. 2016; Silva et al. 2017). Microscopically, they are recognisable by the arrangement and cell type within the tumour itself (Morabito et al. 2023; Ji-Hang et al. 2024). There are three types of tumour, which can be distinguished by their appearance and cell content. The typical histological characteristic of a schwannoma is a tumour well demarcated from the surrounding capsule and arranged in storiform intertwining rosettes, including the presence of fascicular formations of Schwann cells. The perineurioma has a finer appearance consisting of a plexiformly arranged population of perineurial cells. Neurofibromas are more complex in appearance with the presence of both a population of fibroblasts and the production of a fibrous component that arranges in uncircumscribed trabecular, or reticular, formations of the neural component. It can be said that, in veterinary histopathological practice, terminologically, there is also a merging of the above-mentioned units into a single group of BPNSTs. However, some tumours, histologically, show a mixed phenotype of BPNST subunits. In human histopathology, tumours within a single lesion have been described as intermingling in this sense (Feany et al. 1998). Such forms are characterised as a hybrid group of peripheral nerve tumours (HPNSTs). Herein, we assume that similar types of tumours are also found in veterinary medicine, and we believe that their background, including their genetic basis and their behaviour, would be useful to compare with other PNSTs. In our work, based on a case from our practice, we describe the finding of a schwannoma of the mammary gland of a dog, the arrangement of which mimics the transformation into a hybrid peripheral nerve tumour, also with a regressively altered part and regressing mammary alveoli.

## Case description

A Crossbreed 8-year-old, female dog with a tumour in the mammary gland area was brought to the veterinary clinic. An investigation documented one 4 cm large formation on the left mammary gland bar in the caudal region. It was a solid tumorous formation which protruded partially through the skin with some irregularities to the touch. The rest of the mammary gland remained unchanged. Surgery was planned, and carried out under full anaesthesia and the tumour was completely removed, including the peripheral tissue and fixed with a 10% formalin solution and preserved for 24 hours. The laboratory received a tumorous formation of approximately 4 × 3 × 2 cm partially covered by skin. It had a homogenous structure consisting of pale-coloured rigid elastic tissue. Two samples were taken from the tumour for further histopathological investigations.

## Histological and immunohistochemical analysis

The material was analysed using standard histological methods. Three to five μm-thick slices were cut from each sample to a Superfrost^TM^ Microscope Slides Ground 90° Pink Tab (Epredia, Shanghai, China). These slices were stained with haematoxylin-eosin (Bamed, České Budějovice, Czech Republic). The second slices were stained for the immunohistochemistry. Before immunostaining, heat-induced antigen retrieval was performed for 20 min using a pressure cooker (AVAIR IDA, Nitra, Slovak Republic), with a pH 6.0 buffer (Target Retrieval Solution, Low pH; DAKO, Agilent, Cheadle, UK). After this, the slices were allowed to cool and then incubated at room temperature with several primary antibodies, including, Monoclonal Mouse Anti-Human CD56 Clone 123C3 (CD56; Agilent, Carpinteria, USA), Monoclonal Mouse Anti-Human Cytokeratin 7 Clone OV-TL 12/30 (CK7; Agilent, Carpinteria, USA), Monoclonal Mouse Anti-Human Cytokeratin 20 Clone K_S_20.8 (CK20; Agilent, Carpinteria, USA), Monoclonal Mouse Anti-Human Oestrogen Receptor α Clone 1D5 (ER; Agilent, Carpinteria, USA), Monoclonal Mouse Anti-Human Ki-67 Antigen Clone MIB-1 (Ki-67; Agilent, Carpinteria, USA), Monoclonal Mouse Anti-Human Neuron-Specific Enolase Clone BBS/NC/VI-H14 (NSE; Agilent, Carpinteria, USA), Anti-S100 antibody 4C4.9 (S-100; Abcam, Cambridge, UK) and Monoclonal Mouse Anti-Vimentin Clone V9 (VIM; Agilent, Carpinteria, USA). For the washing of the slices, a conventional wash buffer was used (Agilent, Cheadle, UK). For visualisation, an LSAB+System HRP kit (streptavidin-biotin peroxidase detection kit; Agilent, Cheadle, UK) was applied according to the manufacturer’s instructions. The reaction was visualised with a DAB+chromogen kit (Liguid DAB+Substrate Chromogen System; Agilent, Cheadle, UK). Finally, the slices were stained with Mayer haematoxylin (Bamed, České Budějovice, Czech Republic). The samples were described and evaluated in a light-microscopic picture using an Olympus BX53 optical microscope (Olympus, Tokyo, Japan).

## RESULTS

**Figure 1:**
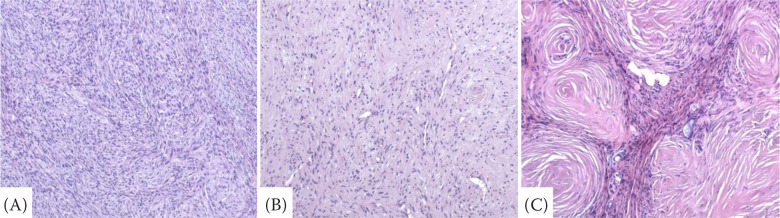
View of the more concentrated (A) and finer (B) part of the tumour, and the part undergoing regressive changes (C) Haematoxylin-eosin 100 ×

**Figure 2:**
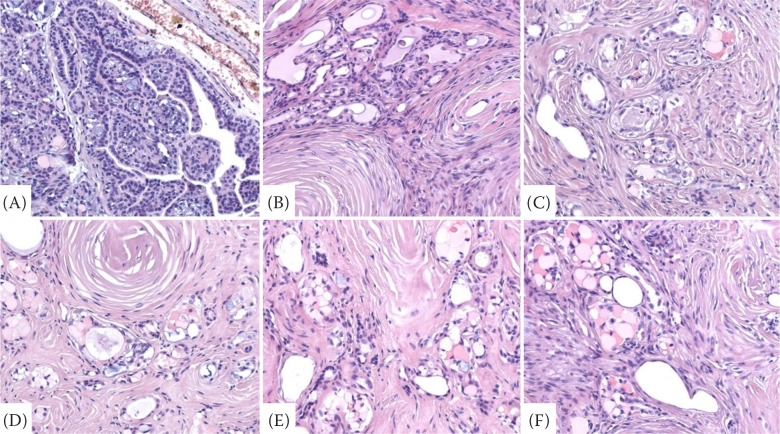
View of the surrounded tumour (A), tumour-involved (B) and tumour-permeated glandular formations undergoing regressive changes (C,D) with prominent inflation of the epithelial lining with cells containing some pinkish secretion (E, F) Haematoxylin-eosin 200 ×

**Figure 3:**
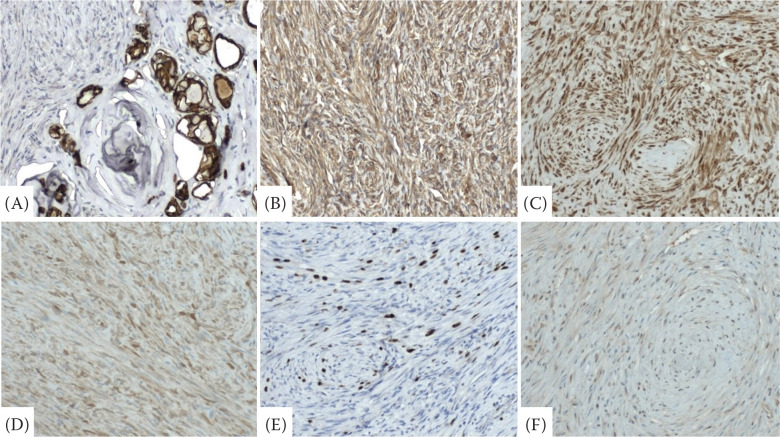
Immunohistochemical profile of the tumour (A) CK7; (B) VIM); (C) NSE; (D) S-100; (E) Ki-67; (F) CD56; A–F 200 ×

Histologically, there was a benign tumour growing expansively and enclosed on one side by a thin fibrous septum, which is formed via the arrangement of two different areas. The first part was arranged in two uncircumscribed, intermingling components. The more concentrated part with a predominance of cellular components, had an alternating variable size, with sometimes compressed, vortical formations that were surrounded by fascicular trabeculae, or interlocking septa with the presence of spindle-shaped elements (Figure 1A). A more vaguely circumscribed, optically finer, portion with a predominance of filamentous components over cellular components was arranged plexiformly consisting of a few spiral-shaped, to spindle-shaped, cells with a single, darker, nucleus shaped like cytoplasm (Figure 1B). The second part was predominantly loose, consisting of variably sized vortical formations composed of coiling thicker eosinophilic lamellae with occasional spindle-shaped elements (Figure 1C). Most lamellae underwent regression with basophilia grading with hyalinisation even with light spacing mimicking loosening the target formations. In contact on a background of somewhat thinned shorter septa of finely pinkish material, individual or few clusters of spiral-shaped to elongated cells were visible. In places, the tumour surrounded or even enclosed groups of apparently normal mammary alveoli (Figure 2A). Elsewhere, it intercalated among groups of alveoli, which underwent regressive changes with the prominent inflation of the epithelial cells by a pinkish material and the degeneration or disintegration of the glandular epithelium (Figure 2B–F). Immunohistochemically, the tumour cells showed intense positivity in the vimentin (VIM) reaction, as well as clear positivity in the neuron-specific enolase (NSE) reaction, variable expression of S-100 protein, and subtle CD56 positivity. Accentuated proliferative activity was assessed by Ki-67. The tumour cells were negative for evidence of cytokeratins (Ck7, Ck20), and oestrogen receptors (ERs) (Figure 3A–F).

## DISCUSSION

Mammary gland tumours of dogs are a group of frequently sent samples to veterinary histopathology laboratories. One paper reported that, in the case of bitches, this is the most frequent type of tumour (Canadas et al. 2019). At the same time, the results of some studies document that benign tumours predominate over malignant tumours (Litterine-Kaufman et al. 2019), while, in other works, this ratio is reported to be reversed (Srisawat et al. 2012), or even a similar frequency of benign and malignant mammary tumours has been reported (Salas et al. 2015). Under the auspices of the World Health Organization, an international histological classification of mammary gland tumours of dogs and cats has been developed, which divides them into several basic groups (Hampe and Misdorp 1974; Misdorp et al. 1999). In this classification, tumours or tumour-like lesions are categorised as hyperplasia/dysplasia, then malignant, benign, and finally unclassifiable tumours. Although epithelial tumours clearly predominate, the finding of tumours of mesenchymal, or even of mixed origin are not rare. Several studies have been published in the context of mammary tumours, which we can understand as an attempt to extend the WHO’s recommended classification, and then as a professional report of our own experience with these tumours.

The work of Goldschmit et al. (2011) compares the histological classification schemes of canine mammary tumours between 1974 and 1999 and proposes a new classification system based on eight groups. Other similarly formulated works are devoted to the diagnostic and prognostic indicators of canine mammary tumours or they are preferentially related to experimental laboratory animals (Cassali et al. 2011; Rudmann et al. 2012; Do Carmo Silva et al. 2019). As they preferentially include primary mammary epithelial tumours, tumours from the peripheral nerve sheath are not mentioned. The mammary gland consists of glandular parenchyma and interstitium, which is composed of richly vascularised sparse connective tissue, univacuolar fat cells, lymphatic vessels, and nerve endings (Makovicky et al. 2013). It is an organ that dynamically undergoes developmental stages depending on the age and depending on the individual physiological changes, even with a different microscopic picture. Here, changes occur in both the glandular and fibrous components (Makovicky et al. 2023). Although our finding is reportedly rare, due to the lower use of special immunohistochemical methodologies in routine veterinary biopsy practice, this may, in fact, not correspond to reality. Based on this fact, a proportion of vaguely differentiated mammary gland tumours could also correspond to tumours originating from neural tissue. Thus, it could also be an underdiagnosed group as evidenced by the findings of neuroendocrine differentiation in canine mammary carcinomas when Chromogranin A, and possibly Synaptophysin, are proven (Nakagaki et al. 2021). In human pathology, both schwannoma and perineuriomas have been diagnosed in the mammary gland, which categorically fall into the group of other tumours and tumour-like conditions (Tan et al. 2014). However, within the canine mammary gland, these are rarer tumours. In one work based on a series of 9 936 canine mammary gland tumours, the spindle-cell lesion group comprised only 1.09% of the canine mammary gland, of which 21 cases were concluded as PNSTs in a review staining, but which were further reduced to 2 cases after additional immunohistochemical staining (Alonso-Diez et al. 2019). In another study, preferentially focusing on canine PNSTs, not a single case was confirmed in the mammary gland based on 70 samples (Boos et al. 2015). Another paper reported that they were diagnosed around the mammary gland in dogs (Ko et al. 2014). Thus, the tumour reported by us is peculiar in two respects: by its localisation and by its arrangement. It is a benign tumour, but, at first glance, it could also mimic malignant forms of tumour arising from the epidermis of the character of a squamous cell carcinoma. In the looser parts, the vortical formations partially resemble keratin pearls, but, immunohistochemically, they are negative for cytokeratins. In addition, the regressing alveoli are strikingly reminiscent of sweat glands in places. Misdiagnosis leads to an inappropriate, ineffective, treatment which could cause secondary damage to the health as well as economic harm. On the one hand, the tumour penetrates the mammary gland parenchyma, but on the other hand, it grows expansively with clearly demarcated borders between the tumour and the adjacent mammary gland. Part of the tumour undergoes regression, with the original spindle-shaped formations taking on the appearance of target-like formations with degenerating lamellar formations. In the more central deeper parts of the tumour, there is an unbounded alternation of more concentrated parts with finer parts. This arrangement could predispose it to the group of so-called hybrid tumours containing a part that corresponds to a schwannoma with indicated plexiform features, and a part that more closely resembles a perineurioma. The regressively altered part is located on the original mammary interstitium, which could have some influence on both the tumour arrangement and its functional characteristics. Indeed, the preserved glandular component is, similarly, subject to regressive changes, which are not commonly found in the normal mammary gland. In one of our studies, we have shown that the fibrous component changes under the influence of the immune system in the tumour environment, and thus it is possible that both the preserved glandular component and the tumour regress under the influence of the dynamically changing mammary gland parenchyma (Caja et al. 2021). On the other hand, the degeneration of the alveoli themselves may be induced by compression of their ducts or circulatory system by the tumour. The hormonal influence in the mammary gland is similarly substantiated, but we also mention that ER positivity has not been reported. Ultimately, these types of tumour should not cause diagnostic difficulties, but they should be clearly differentiated from malignant epithelial mammary tumours and epithelial tumours of the skin, which are prognostically worse.

Here, using a case report of a peripheral nerve sheath tumour from practice, we document its microscopic arrangement and compare the findings with similar cases documented in the literature. An unusual hybrid peripheral nerve tumour concluding like Schwannoma in mammary gland of the dog is presented. Despite the uncommon occurrence of PNSTs in the mammary gland, it is possible that this is an underdiagnosed entity confused with other types of mesenchymal tumours, possibly because of its variable arrangement. The finding opens the discussion of the possible influence of the mammary interstitium on the arrangement and redistribution of the cells of tumours, which may thus occupy preferential locations within the mammary anatomy.

It might be a suggestion to include hybrid tumours as part of a new classificatory system within the WHO mesenchymal tumours classification scheme and include the tumours that occur in the mammary glands of dogs and cats.
